# Supplementary material for: Modelling Skylarks (Alauda arvensis) to Predict Impacts of Changes in Land Management and Policy: Development and Testing of an Agent-Based Model
Source: PLoS One. 2013 Jun 6;8(6):e65803. doi: 10.1371/journal.pone.0065803 (PMC3675089; doi:10.1371/journal.pone.0065803)
Supplement: Supporting Information S4 — The skylark ODdox as a zipped archive. (ZIP) [file pone.0065803.s004.zip › Skylark_ODdox/classsk_t_territory-members.html]

ALMaSS Skylark ODdox: Member List


ALMaSS Skylark ODdox
 2.0


- Main Page
- Related Pages
- Classes
- Files

- Class List
- Class Index
- Class Hierarchy
- Class Members

skTTerritory Member List

This is the complete list of members for skTTerritory, including all inherited members.

|  |  |  |
| --- | --- | --- |
| F\_Owner | skTTerritory |  |
| GetQuality(void) | skTTerritory | inline |
| GetVirtualDiameter() | skTTerritory | inline |
| m\_competitionscaler | skTTerritory |  |
| m\_heterogeneity | skTTerritory | protected |
| m\_hr\_polys | skTTerritory |  |
| m\_hr\_sizes | skTTerritory |  |
| m\_Location\_x | skTTerritory |  |
| m\_Location\_y | skTTerritory |  |
| m\_nest\_pos\_validx | skTTerritory |  |
| m\_nest\_pos\_validy | skTTerritory |  |
| m\_nest\_valid | skTTerritory |  |
| m\_polys | skTTerritory |  |
| m\_Quality | skTTerritory | protected |
| m\_range\_div10 | skTTerritory |  |
| m\_sizes | skTTerritory |  |
| m\_VirtualDiameter | skTTerritory | protected |
| m\_x\_div10 | skTTerritory |  |
| m\_y\_div10 | skTTerritory |  |
| Owner | skTTerritory |  |
| SetNestPossibility(bool valid, int nx, int ny) | skTTerritory | inline |
| SetQuality(int a\_qual) | skTTerritory | inline |
| SetVirtualDiameter(double dia) | skTTerritory | inline |
| Size | skTTerritory |  |
| skTTerritory(int x, int y, int TheSize, int TheQuality, int a\_x\_div10, int a\_y\_div10, int a\_range\_div10) | skTTerritory |  |
| TestNestPossibility() | skTTerritory |  |


- Generated on Thu Jan 10 2013 13:15:37 for ALMaSS Skylark ODdox by
   1.8.1.1
